# Social behavior in the “Age of Empathy”?—A social scientist's perspective on current trends in the behavioral sciences

**DOI:** 10.3389/fnhum.2013.00236

**Published:** 2013-05-31

**Authors:** Svenja Matusall

**Affiliations:** MINDLab and Interacting Minds Centre, Aarhus UniversityAarhus, Denmark

**Keywords:** social neuroscience, prosocial behavior, history of neuroscience, epistemology, science studies

## Abstract

Recently, several behavioral sciences became increasingly interested in investigating biological and evolutionary foundations of (human) social behavior. In this light, prosocial behavior is seen as a core element of human nature. A central role within this perspective plays the “social brain” that is not only able to communicate with the environment but rather to interact directly with other brains via neuronal mind reading capacities such as empathy. From the perspective of a sociologist, this paper investigates what “social” means in contemporary behavioral and particularly brain sciences. It will be discussed what “social” means in the light of social neuroscience and a glance into the history of social psychology and the brain sciences will show that two thought traditions come together in social neuroscience, combining an individualistic and an evolutionary notion of the “social.” The paper concludes by situating current research on prosocial behavior in broader social discourses about sociality and society, suggesting that to naturalize prosocial aspects in human life is a current trend in today's behavioral sciences and beyond.

## Introduction

Recently, several behavioral sciences, for instance neuroeconomics (e.g., Fehr and Fischbacher, 2003), primatology (e.g., De Waal, 2009) and social neuroscience (e.g., Frith and Frith, 2010), became increasingly interested in investigating biological and evolutionary foundations of (human) social behavior. Scholars from these fields argue that the biology of humans is itself much more prosocial than previously thought. Prosocial behavior is a core element of human nature. It is rooted in each individual, has evolved during the course of evolution, is located in the brain, its genes, functions, hormones and neurotransmitters and is embedded in an environment. A central concept of this new perspective on human nature is the “social brain” (Brothers, 1990) that is not only able to communicate with the environment but rather to interact directly with other brains via neuronal mind reading capacities such as empathy (see Young, 2012a).

Taking social neuroscience as an example, this paper explores the notion of “social” in contemporary behavioral sciences and how a new concept of human nature emerges. At the core of this new concept is the notion that default human behavior is prosocial. The paper sets out to investigate what “social” means in social neuroscience. (1), the research field is introduced before a glance in the history of the social sciences shows that “social” is by no means an unambiguous term (2). The historical roots of the social brain are explored (3) and the paper concludes (4) by situating current research on social behavior in broader discourses about sociality and society, suggesting that the trend to look for prosocial aspects in human life, culture and society also takes place in other spheres of society.

## What is social neuroscience?

Social neuroscience is much more diverse than this brief perspective paper could picture and hence this paper's aim can only be to outline general trends within the field. The term “social neuroscience” was first coined by social psychologists Gary Berntson and John Cacioppo in 1992 (Cacioppo and Berntson, 1992). They propose a cooperation between social psychology and neuroscience in order to avoid the pitfalls of reductionism by adding multiple perspectives to given problems. But it took another decade before a field with research groups, professorships, university courses, textbooks, conferences, societies, and journals emerged that calls itself social neuroscience (Matusall et al., 2011). In this process, a second important impetus came from a paper by Ochsner and Lieberman (2001), who should also be named among the founding figures of the field.

Many of social neuroscience's topics of interest fall into the realm of classic social psychology, for instance the study attitudes, prejudices and stereotypes (Matusall, 2012). Interestingly, however, is the field's new focus on emotion, empathy and altruism (cf. Decety and Ickes, 2009; Singer and Lamm, 2009). Recently, prosocial behavior moved into the center of attention, not only in social neuroscience but also in other behavioral sciences such as primatology and anthropology (cf. De Waal, 2009; Tomasello, 2009).

## What does social mean in social neurosciences?

In social neuroscience, prosocial behavior is sought in genes, brains and evolutionary past. “Social” is simultaneously understood as a capacity of the organism's brain to cope with the environment and as an evolutionary advantage of the species. This perspective on the social differs fundamentally from sociology's perspective, where the social can be anything from the sum of individual actions to power relations or social structures. The list of phenomena having been defined to be social in the course of the history of the social sciences is rather long and diverse as (Greenwood, 1997, p. 3) points out by giving a random collection of those phenomena: “states, families, armies, religious organizations, literary societies, mobs, street brawls, people chatting on a street corner, the Roman Catholic Church, the Renaissance, insect communication, dominance hierarchies among primates, language, financial instruments, and traffic flow in a city.” Thus, “social” is by no means an unambiguous term and for understanding social neuroscience's notion of “social,” it is crucial to look into the history of experimental social psychology, which is one of social neuroscience's intellectual parent disciplines. Looking at the questions social neuroscientists tackle in their research, it soon becomes evident that they focus on the way social stimuli are perceived and processed in the brain—no matter whether they study empathy, attitudes toward out-group members or voters' behavior. This individual-centered approach may be self-evident for social neuroscientists, yet it is a historically contingent approach as will be shown in the next section.

## Genealogy of a concept

The individualistic perspective on the social has a long tradition in experimental social psychology: since its emergence in the 1920's, this discipline has understood itself as a branch of individual psychology (Allport, 1924), investigating whether and how the perception and processing of social stimuli differed from the perception and processing of non-social stimuli. In order to apply experimental methods to such questions, social psychologists had to frame their objects of investigation as statistically measurable. In this process, the social was redefined as a quality of countable entities. This perspective differed from theories in 19th century social psychology that connected the social with morality and religion, respectively with institutionalized power (Danziger, 1997). Moreover, the individualist notion of the social had a crucial role in defining and defending the individualistic American Way of Life against collectivist notions of society and the individual (Rose, 1998). The political background of its emergence seems all but forgotten by those employing this notion of social today as a variable investigated by experimental methods. Most social neuroscientists are trained in social psychology and most positions are located in psychology departments. Their research questions and their argumentation stand in the tradition of experimental social psychology. By relocating the “social” in the individual's brain and neurobiology, social neuroscientists are in line with their predecessors in treating it as an individual capacity.

## Perspectives from sociology

Looking with the eyes of a sociologist, investigating problems in small pieces, such as brain activation, entails the risk of losing the perspective on the broader picture and taking the small piece for the whole problem (Star, 1983). The experimental design of “social” in social neuroscience research requires rendering research in a quantitative fashion.^1^ This does not necessarily imply a reduction of complexity in the stimuli presented but in the questions asked. If complex issues such as voters' emotional reactions to election outcomes or empathy with members of an “out-group” are measured by quantitative tools, it has to be assumed that complex phenomenona can be split up into several problems and thus are not more than the sum of their parts. This approach differs fundamentally from hermeneutic approaches towards complex phenomena, which are more interested in meaning than in mechanisms and which are dominant in humanities and non-quantifying social sciences.

To some extent, social neuroscientists seem to be aware of this and pay credit to the problem of complexity by drawing on the notion of levels (Cacioppo and Berntson, 1992; Ochsner and Lieberman, 2001). Cacioppo and Berntson (1992) maintain that although the brain is an essential component of all social beings, brain, behavior and society are each too complex to be reduced to one another. Hence, social neuroscience aims to combine data generated on different levels to reach a better comprehension of social behavior. Yet, knowledge from other disciplines can only be integrated if compatible with the standards of quantifying sciences and qualitative knowledge is difficult to incorporate in such paradigms.

## History of the social brain^2^

Not only in social psychology, also in the brain sciences, questions about the “social” have a long tradition. The relationship between the brain and the social has been an issue of hot debate ever since the emergence of modern brain science in the late 18th century. In these debates, the pendulum has been swinging happily back and forth between seeing either nature or nurture as responsible for human behavior. Early 19th century's phrenologists, for instance, defined a cerebral faculty for each human property and thus saw a clear causal direction from brain to behavior, while psychiatrists in the second half of the 19th century made harmful social conditions responsible for psychiatric disorders and thus reversed causal directions (Hagner, 2007). Theories of evolution were central to 19th and early 20th century's concepts of the brain and the social. These theories were associated with a hierarchical organization of brain areas: the younger, more evolved parts such as intellectual capacities or morality controlled older parts such as drives and emotions (e.g., Jackson, 1884).

Not least as a reaction to the role medicine and biological sciences played in Nazi ideology, after the Second World War research in the West was dominated by behaviorism, cybernetics and cognitive science (Hagner, 2007). During that time questions about human interactions did not play a role in mainstream neuroscience and psychology. This began to change slowly in the 1980's and with even more force in the 1990's when the social brain returned to the debate in three independent theories about the relationship between brain and social: the *social brain hypothesis*, the *somatic marker hypothesis* and the *mirror neuron theory*, which will be discussed in next section.

## The social brain since the 1990's

The social brain hypothesis suggests that the size of the neocortex and the group size of mammals living in social groups correlate (Brothers, 1990; Dunbar, 1998). The bigger the group, the more complex the social situations which the brain has to process. Certain cognitive skills evolved to cope with social complexity. Consequently, the way we act in social interactions is determined by evolutionary heritage. The social brain hypothesis does not explicitly discuss the impact of history, culture, society, or life experiences on social cognition abilities in an individual or a group. Only in an evolutionary time frame these factors may have an impact on how future generations may engage with each other (Matusall, 2012). Nor does it answer the “hen and egg” question of whether the complex social groups or the cerebral capacities for processing them was first; or whether both evolved together. What it does is providing an evolutionary explanation for both, human sociality and the species' big brains.

The second theory, the somatic marker hypothesis was introduced by neuropsychiatrist Antonio Damasio and it suggests that positive experiences are connected with positive memories leaving a positive somatic marker, i.e., an incentive for deciding in favor of similar actions in future decision-making processes while negative experiences are connected with negative memories leaving negative a somatic marker, i.e., an alarm bell, leading to deciding against similar actions in future decision-making processes. These markers are acquired during socialization not only through experienced events but also by incorporating norms and rules and can change throughout life if new experiences occur (Damasio et al., 1991). This means a crucial shift in thinking about the social and the brain, which is later taken up by social neurosciences and related disciplines (Cacioppo and Berntson, 2005; Glimcher et al., 2009; Ariely and Berns, 2010). The somatic marker hypothesis couples biology with cultural and social environments. Somatic markers and thus the ability to act socially is part of the biological make-up with which humans are born, yet the way this sociality takes shape depends on the particular beliefs and values of the society one is born into (Damasio, 1994).

Around the same time when Damasio developed his somatic marker hypothesis, in Italy a team of neuroscientists reported to have found a neural basis of the capacity of primates to engage with others (di Pellegrino et al., 1992). It followed an ever-increasing interest in these neurons, which were soon named mirror neurons, and their hypothesized function included a growing number of areas of social life (e.g., Gallese, 2003). This theory did not only seem to explain human social behavior, development and learning but also how we participate, for example, in another person's joy and distress automatically, by biological default. Yet, after the first excitement faded away, mirror neurons became contested (see for instance Hickok, 2008; Gallese et al., 2011) and it is too early to decide whether the mirror neuron theory will become canonical knowledge in the attempt of how mind and brain work. Like other such theories such as the concept of brain plasticity, mirror neuron theory enjoys a broad popularity outside the scientific community—perhaps not least because it provides a biology based on prosociality. The idea of biologically automatic responses to other people's behavior and even emotions is alluring, since it seems to argue in favor of a prosocial default of human nature. Even though feeling does not automatically lead to acting, being able to empathize may lay a foundation for prosocial action.

These three theories and their focus on social aspects of the human condition differ from preceding notions of human nature in one fundamental respect: Homo sapiens are understood as a social and empathic species rather than an individualistic one. Contrary to older models, it is now suggested that it comes quite naturally to humans to act prosocially. Evidence for the prosocial nature of humankind is found in humans' evolutionary history and the neurobiological and hormonal substrate of the brain. By looking at social behavior from this perspective, it appears that cooperation and altruism are beneficial. Working together, so the argument goes, made life easier and increased the chances of survival of the group's offspring (see e.g., Brothers, 1990 and Dunbar, 1998).

## Future perspectives

Evolutionary reasoning about prosociality can be summarized as follows: since Homo sapiens are a social species, organized in communities, individuals, who are able to decipher social stimuli and to act in prosocial ways had better chances of reproduction and hence, social brains evolved.^3^ This evolutionary heritage equips contemporary humans with the tools for coping with the complexity of social organizations and to engage in social relationships. Not everyone acts prosocially all the time, but every healthy person bears in themselves the potential to do so and has the option to act on that potential. This perspective on sociality means a shift in the conceptual framework of what it is the norm and what needs explanation. While protagonists of this new version of human nature do not deny that aggression is as much part of human nature as is empathy, it now becomes marked as the other, the trait which needs to be explained and this also provides a new perspective on pathologies such as psychopathy or autism, which are now defined by their lack of empathy (e.g., Baron-Cohen, 2011; Blair, 2011). But not only pathologies, even everyday behavior such as envy is interpreted in terms of empathy, respectively the lack thereof (e.g., Shamay-Tsoory, 2009). This does not mean that antisocial behavior is no longer a part of this paradigm. Yet, it becomes the other, the non-normal, which needs to be explained.

In social neuroscience, the individualistic notion of social rooting in American social psychology and the more collectivist notion of the social rooting in anthropology come together and thus in this framework, social relations are intelligibly investigated within the individual. The focus is not on structures, institutions, power relations, all things that can potentially be changed, but on the social as a biological category—nature—that cannot be changed. Sociality becomes a naturalized, innate quality and thus every “normal” individual is capable of behaving prosocially. At a time when responsibility for social cohesion is de-centralized, the neural capacity for prosociality is found.

## Neosociality?

Social neuroscience's notion of social relates to a new notion of what human beings are and how they normally act, in short a new version of a biologically based human nature. In this narrative, sociality is the driving force behind human evolution.

The notion of “social” employed in social neuroscience research is located in the individual brain, its ability to decode a certain kind of stimuli and to interact with others. It is a noteworthy historical concomitance that the investigation of social interactions via social structures or collective processes is replaced by the investigation of processes that take place within individuals at the same time when, in a broader societal setting, collectivist solutions have been replaced by more individual solution (e.g., in welfare, see for instance Sennett, 2006; Lessenich, 2008). Rabinow (1999) described this development as the transformation towards a “biosociality”—social structures become less important while identities are more and more based on individual (i.e., genetic) attributes than on social or group attributes. Investigating the social via communal genetic make-up or individuals' brains is rather different from studying the external conditions for a social structure. In this approach, prosocial behavior becomes something innate and thus every normal individual is capable of behaving prosocially.

## Conclusion

Social neuroscience is an interdisciplinary endeavor aiming to investigate sociality. Taking its methods from social psychology and cognitive neuroscience and its explanatory frame from evolutionary anthropology, it defines the social as both a feature of Homo sapiens' environment and an inherent human capacity to cope and survive. Doing so, it contributes to a new, prosocial notion of human nature. The lens through which social behavior is studied, has changed.

Yet, at the moment, both its focus on quantitative methods and reservations from many arts and social sciences exclude qualitatively operating social science from participating in this endeavor. A methodological and epistemological openness on both sides would be desirable because this could really increase knowledge about social conditions of human nature. Examples for such openness and collaborations can for instance be found in projects on “neurofeminism” (Bluhm et al., 2012; Dussauge and Kaiser, 2012; Einstein, 2012; Matusall, in press). These projects experiment with collaborations bridging the gap between qualitative and quantitative disciplines.

### Conflict of interest statement

The author declares that the research was conducted in the absence of any commercial or financial relationships that could be construed as a potential conflict of interest.

## Footnotes

^1^On the potential dangers of the “mereological fallacy”, see Bennett and Hacker, 2003 and also Krüger, 2010.

^2^For more detailed historical analyses of discourses on the social brain and its relationship to society, see the recent work by anthropologist Allan Young: Young, 2011, 2012a,b. For a philosophical perspective on prosociality in neuroeconomics, and particularly a critical examination of the notion of altruistic punishment, see e.g., Klein, 2012.

^3^The relationship between prosociality, cooperation, and altruism is complex and by no means uncontested in evolutionary psychology and other behavioral sciences. For overviews over the debate see e.g., Henrich and Henrich, 2006; Boyd and Richerson, 2009.
